# Incremento de las concentraciones de procalcitonina en pacientes intoxicados por paracetamol

**DOI:** 10.1515/almed-2020-0081

**Published:** 2020-11-13

**Authors:** Luis García de Guadiana-Romualdo, Carlos Rodríguez Rojas, Verónica Ramos Arenas, Rubén Cárdenas Gámez, María Dolores López Abellán, Mercedes González Morales

**Affiliations:** Servicio de Análisis Clínicos, Hospital Universitario Santa Lucía, C/ Mezquita, s/n, Paraje Los Arcos, Santa Lucía, Cartagena, 30202, Cartagena, España

**Keywords:** paracetamol, intoxicación, procalcitonina

## Abstract

**Objetivos:**

La intoxicación por paracetamol es una de las causas no infecciosas asociada al incremento de la concentración de procalcitonina. Presentamos dos casos de pacientes ingresados por intoxicación por paracetamol en el que los datos analíticos revelaron un aumento significativo de procalcitonina sin datos clínicos, radiológicos y/o microbiológicos de infección. El mecanismo que explicaría este incremento no ha sido aclarado.

**Caso clínico:**

Se presentan dos casos correspondientes a dos pacientes atendidos en el Servicio de Urgencias por intoxicación por paracetamol requiriendo ambos el ingreso en la Unidad de Cuidados Intensivos (UCI). Ambos mostraron un incremento de procalcitonina en las primeras horas de ingreso, sin evidencia clínica y/o microbiológica de infección que justificase dicho incremento. De forma llamativa, sólo el paciente 1 presentó disfunción hepática, con alteración de las concentraciones de alanino aminotransferasa (ALT), aspartato aminotransferasa (AST), bilirrubina y bilirrubina esterificada, hallazgos no observados en la paciente 2.

**Conclusiones:**

los pacientes presentados en este caso clínico mostraron un aumento de procalcitonina asociado a la intoxicación por paracetamol, pero sólo uno de ellos mostró signos de disfunción hepática. Ello sugiere que en la intoxicación por este fármaco la secreción del biomarcador no estaría solamente ligado al daño sobre el hepatocito, sino que otros mecanismos en los que estarían implicados otros órganos y tejidos contribuirían al aumento de procalcitonina. En cualquier caso, y aunque el mecanismo no ha sido perfectamente aclarado, es importante conocer esta limitación para el uso de procalcitonina como marcador de infección en pacientes intoxicados por paracetamol.

## Introducción

La procalcitonina es un marcador de gran interés para el diagnóstico de las infecciones bacterianas graves y la monitorización del tratamiento antibiótico [1], [2]. También se han descrito incrementos asociados al carcinoma de células C, pacientes quemados o traumatológicos [3] e intoxicaciones por drogas de abuso como las anfetaminas [4] o fármacos como el paracetamol [5], sin evidencia de infección bacteriana. En este último caso, el mecanismo de la elevación no ha sido aclarado y podría estar relacionado con la implicación de otros órganos, además del daño celular hepático [6], [7], [8].

Presentamos dos pacientes atendidos en el Servicio de Urgencias (SU) por intoxicación por paracetamol, que mostraron un incremento de las concentraciones de procalcitonina sin evidencia clínica o microbiológica de infección bacteriana. El primer paciente mostró un aumento significativo de los marcadores bioquímicos de función hepática, hallazgos no observados en el segundo paciente. Esta diferencia sugiere que además del daño hepático inducido por el paracetamol, otros mecanismo contribuirían a incrementar los niveles de procalcitonina [5].

## Casos clínicos

### Caso 1

Paciente varón de 65 años que ingresó en el SU por descenso del nivel de consciencia tras la ingesta en las 6 horas previas de bromazepam, zolpidem y alcohol, en cantidades no conocidas, con finalidad de autolisis. En sus antecedentes personales, destacaban el consumo crónico de alcohol y dos episodios de flutter común, que requirió ablación del istmo cavo-tricuspídeo y anticoagulación con acenocumarol y apiroxaban. Al ingreso en el SU, los signos vitales fueron los siguientes: presión arterial: 163/94 mm Hg, frecuencia cardiaca: 66 latidos/min; frecuencia respiratoria: 19 respiraciones/min, temperatura: 36 °C; y puntuación en la escala Glasgow: 15. En los resultados analíticos al ingreso, destacaba un incremento de etanol en sangre y un resultado negativo para drogas de abuso en orina, incluyendo benzodiacepinas, sin otros hallazgos significativos (Tabla 1). Se inició tratamiento con flumazenilo, apreciándose una mejoría significativa de la capacidad sensorial. Reevaluado el paciente por el Servicio de Psiquiatría, declaró haber ingerido aproximadamente 20 g de paracetamol, por lo que se solicitó al laboratorio la medida de niveles de dicho fármaco en la primera muestra de sangre extraída, con un resultado de 162,4 μg/mL, tras el cual se inició tratamiento con N-acetil cisteína, que se mantuvo durante 72 horas, y sin alteraciones significativas de los biomarcadores de función hepática (ALT, AST y bilirrubina). Desde el laboratorio, y dada la descripción de casos anteriores observando incrementos de procalcitonina en pacientes intoxicados por paracetamol, se midió también su concentración sérica, sin alteración significativa de la misma.

**Tabla 1:** Hallazgos de laboratorio en el caso 1.

	Intervalo de referencia	Tiempo_admission_ Ingreso en SU Día 1	Tiempo_12h_	Tiempo_18h_	Tiempo_24h_ Ingreso en UCI Día 2	Tiempo_48h_ Día 3	Tiempo_72h_ Día 4	Tiempo_96h_ Día 5	Tiempo_168h_ Alta UCI Día 6	Tiempo_240h_ Día 8	Tiempo_264h_ Día 9	Tiempo_336h_ Día 12 Exitus
Paracetamol, μg/mL	<5^a^	162,4	51,8	18,6	6,3	–	–	–	–			
ALT, U/L	<41	31	170	319	472	6.988	4.386	2.643	438	210	143	139
AST, U/L	<40	35	248	449	691	11.939	2.277	1.342	59	60	55	–
Bilirrubina, mg/dL	<1,2	0,4	3,2	3,8	3.8	3,7	4,1	4,3	1,9	1,7	2,2	1,2
Bilirrubina directa, mg/dL	<0,3	–	2,3	1,9	2,7	1,9	3,3	3,5	1,7	1,3	1,5	–
TP, ratio	0,9–1,1	–	1,54	2,24	2,53	3,24	3,24	1,81	1,32	–	1,22	1,7
TTPa, ratio	0,9–1,1	–	0,97	1,00	1,03	1,01	1,01	–	1,01	–	0,83	0,84
Procalcitonina, ng/mL	–	0,04	23,15	20,61	21,69	11,84	6,18	4,04	1,97	0,69	0,60	2,45
PCR, mg/dL	<0,5	<0,03	<0,03	0,6	1,0	3,5	5,8	7,4	14,3	18,7	24,3	14,18

SU, Servicio de Urgencias; UCI, Unidad de Cuidados Intensivos; ALT, alanina aminotransferasa; AST, aspartato aminotransferasa; TP, tiempo de protrombina; TTPa, tiempo de tromboplastina parcial activado; PCR, proteína C reactiva. ^a^Concentraciones tóxicas postdosis: - Tras 4 h postdosis: >200. - Tras 8 h postdosis: >100. - Tras 12 h postdosis: >50.

En las analíticas de control, realizadas a las 12 y 18 horas después del ingreso en el SU, se evidenció una disfunción hepática, con aumento progresivo de aminotransferasas, bilirrubina, bilirrubina esterificada y tiempo de protrombina, requiriéndose el ingreso del paciente en la Unidad de Cuidados Intensivos (UCI). Al ingreso en dicha unidad, el paciente presentaba una concentración de paracetamol de 6,3 μg/mL y hallazgos analíticos de empeoramiento de la función hepática. En las analíticas extraídas durante las primeras 24 horas de ingreso se constató la elevación significativa de las concentraciones de procalcitonina, a pesar de no presentar signos y/o síntomas de infección.

Durante el ingreso en UCI, el paciente presentó un episodio de fibrilación auricular revertido con amiodarona y un síndrome de bradicardia-taquicardia que requirió la implantación de un marcapasos definitivo, una buena evolución a nivel respiratorio y neurológico y sin signos y/o síntomas de infección, a pesar de lo cual se mantuvieron elevadas las concentraciones de procalcitonina, aunque en descenso progresivo. Tras una estancia de 7 días el paciente fue dado de alta a Medicina Interna, con un descenso significativo de los marcadores de función hepática.

**Figura 1:**
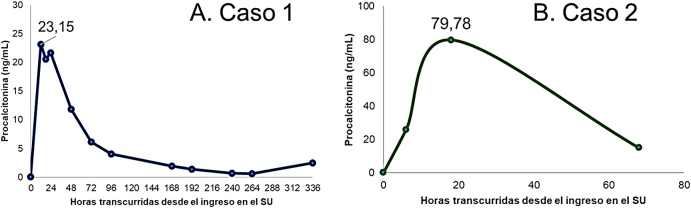
Cinética de la concentración de procalcitonina (PCT) en ambos pacientes. SU, Servicio de Urgencias.

Al ingreso en la planta de Medicina Interna, el paciente presentaba buen estado general, pero durante su estancia presentó una arritmia cardiaca por fibrilación auricular paroxística con empeoramiento radiológico franco, que mejoró tras la administración de furosemida; tras iniciar nuevamente tratamiento con amiodarona el paciente presentó un edema pulmonar bilateral. Las pruebas microbiológicas solicitadas fueron todas negativas (reacción en cadena de la polimerasa para SARS-CoV-2 y virus influenza A/B y antígeno en orina de *Legionella* y *Streptococcus pneumoniae*). Analíticamente, destacaron un incremento progresivo de PCR, ya iniciado en la UCI, y de creatinquinasa, que alcanzó un valor de 7.434 U/L el día del fallecimiento, en el cual la procalcitonina aumentó también de forma significativa, tras un descenso progresivo (Figura 1A). El paciente falleció, tras una estancia hospitalaria de 12 días, por edema pulmonar no cardiogénico de origen no filiado y que pudiera atribuirse a una neumonía nosocomial, no confirmada microbiológicamente, o a una neumonitis asociada a la toxicidad pulmonar de la amiodarona.

### Caso 2

Paciente mujer de 34 años que ingresa en el SU por intoxicación medicamentosa con finalidad autolítica, debido a la ingesta 12 horas antes de lorazepam (12 mg), trazodona (2 g) y paracetamol (38 g). Al ingreso, la paciente estaba normotensa, afebril, eupneica y obnubilada, con una puntuación en la escala Glasgow de 14.

Tras lavado gástrico e inicio de tratamiento con N-acetil cisteína, en la primera analítica el hallazgo más significativo (Tabla 2) fue, además de la concentración de paracetamol (149,6 μg/mL), un resultado positivo para benzodiacepinas en orina, sin datos analíticos de disfunción hepática. Al ingreso en UCI, aproximadamente a las 18 horas tras la ingesta medicamentosa, se inició tratamiento con flumazenilo, se mantuvo durante 24 horas más el tratamiento con N-acetil cisteína y se mantuvo una estrecha vigilancia clínica, electrocardiográfica y analítica. Durante la estancia en UCI, la paciente permaneció estable clínicamente, sin datos analíticos de disfunción orgánica, pero con elevaciones significativas de procalcitonina (Figura 1B), aunque la paciente no presentó ningún dato clínico sugestivo de infección. Tras 3 días de estancia en UCI, la paciente fue dada de alta al Servicio de Medicina Interna, desde donde fue alta hospitalaria dada su evolución favorable.

**Tabla 2:** Hallazgos de laboratorio en el caso 2.

	Intervalo de referencia	Tiempo_admission_ Ingreso en SU	Tiempo_6h_	Tiempo_20h_ Ingreso en UCI	Tiempo_68h_
Paracetamol, μg/mL	<5^a^	149,6	40,0	<5,0	–
ALT, U/L	<33	18	18	16	20
Bilirrubina, mg/dL	<1,2	0,4	0,5	0,5	0,4
TP, ratio	0,9–1,1	1,11	1,35	1,30	–
TTPa, ratio	0,9–1,1	0,80	0,92	–	–
Procalcitonina, ng/mL	–	0,12	25,80	79,78	15,11
PCR, mg/dL	<0,5	<0,03	<0,03	<0,03	4,5

SU, Servicio de Urgencias; UCI, Unidad de Cuidados Intensivos; ALT, alanina aminotransferasa; TP/INR, tiempo de protrombina; TTPa, tiempo de tromboplastina parcial activado; PCR, proteína C reactiva. ^a^Concentraciones tóxicas postdosis: - Tras 4 h postdosis: >200. - Tras 8 h postdosis: >100. - Tras 12 h postdosis: >50.

## Discusión

La procalcitonina es actualmente el biomarcador más útil en el diagnóstico de la infección y predicción de bacteriemia, la valoración de la severidad y la toma de decisiones respecto a la instauración del tratamiento antibiótico, especialmente de la respuesta al mismo, para la desescalada del tratamiento antibiótico [3]. Sin embargo, también se han descrito incrementos de procalcitonina en otras condiciones no infecciosas [3], incluyendo la intoxicación por paracetamol [5], lo que limitaría su utilidad como marcador de infección en estos pacientes, aunque esta capacidad se mantendría en pacientes con fallo hepático agudo no debido a intoxicación por paracetamol [6].

El mecanismo que explicaría dicha elevación no ha sido todavía perfectamente aclarado. En nuestro caso, presentamos dos pacientes en los que se describió dicho incremento, pero con un comportamiento totalmente diferente respecto a la hepatoxicidad inducida por el fármaco, valorada en función de las concentraciones de marcadores de función hepática, que en el caso 2 no mostraron ninguna elevación ni al ingreso en el hospital ni durante la estancia en el mismo.

Recientemente Tschiedel y cols. [7] han propuesto dos posibles mecanismos para explicar el aumento de procalcitonina en la intoxicación por paracetamol. El primero sería la propia hepatoxicidad inducida por el fármaco, que causaría la secreción de ciertas citoquinas, como la interleuquina 2 o el factor de necrosis tumoral alfa, que se comportarían como mediadores para la elevación de la concentración de procalcitonina. Además, un segundo mecanismo estaría relacionado con el papel de otros tejidos como el endotelio. También Lovas y cols. [4], en una intoxicación por paracetamol, sugieren la implicación de otros tejidos dañados e incluso de células del sistema inmune en el incremento de procalcitonina.

El producto responsable de la hepatotoxicidad ligada al paracetamol es la N-acetil-p-benzoquinonimina (NAPQI), procedente del metabolismo oxidativo mediado por enzimas del citocromo P450. Un estudio experimental en ratas demostró que la actividad de dichos enzimas podía ser inducida por la ingesta de paracetamol a dosis no hepatotóxicas y que cierto grado de hepatotoxicidad podría desarrollarse incluso sin elevación de los valores de las aminotransferasas [9]. Tschiedel y cols. [7] han sugerido la implicación en el incremento de procalcitonina asociado a la intoxicación por paracetamol de otros órganos como la célula endotelial a través de la producción de NAPQI.

Finalmente, Ahn y cols. [5] han sugerido recientemente que la ingesta repetida de dosis supraterapéuticas de paracetamol (“*staggered overdose ingestión*”), que no pudo ser confirmada en el caso 2, podría generar un patrón diferente de hepatotoxicidad, sin elevación de los enzimas hepáticos, a la ingesta en una dosis única. Estos autores plantean también la necesidad del análisis de subtipos de procalcitonina, no posible con las metodologías actuales, en pacientes con intoxicación por paracetamol, lo que contribuiría a aclarar las posibles fuentes de secreción de este biomarcador.

## Conclusiones

En los casos expuestos, hemos descrito la elevación de la concentración de procalcitonina en dos pacientes con intoxicación por paracetamol, observada con independencia de las alteraciones analíticas asociadas a la disfunción hepática, y sin evidencia de infección. Más estudios son necesarios para aclarar los mecanismos que explicarían este aumento, pero es necesario conocer esta limitación para el uso de la procalcitonina como marcador para la detección y seguimiento de la infección en pacientes intoxicados con paracetamol.
